# Risk factors for major external structural birth defects among children in Kiambu County, Kenya: a case-control study

**DOI:** 10.12688/f1000research.50738.2

**Published:** 2021-04-30

**Authors:** George N. Agot, Marshal M. Mweu, Joseph K. Wang'ombe

**Affiliations:** 1 School of Public Health, College of Health Sciences, University of Nairobi, Nairobi, Kenya

**Keywords:** Major external structural birth defects, risk factors, case-control study, Kenya

## Abstract

**Background:** Although major external structural birth defects continue to occur globally, the greatest burden is shouldered by resource-constrained countries with no surveillance systems. To our knowledge, many studies have been published on risk factors for major external structural birth defects, however, limited studies have been published in developing countries. The objective of this study was to identify risk factors for major external structural birth defects among children in Kiambu County, Kenya.

**Methods: **A hospital-based case-control study was used to identify the risk factors for major external structural birth defects. A structured questionnaire was used to gather information retrospectively on maternal exposure to environmental teratogens, multifactorial inheritance, and sociodemographic-environmental factors during the study participants' last pregnancies.

Descriptive analyses (means, standard deviations, medians, and ranges) were used to summarize continuous variables, whereas categorical variables were summarized as proportions and percentages in frequency tables. Afterward, logistic regression analyses were conducted to estimate the effects of the predictors on the odds of major external structural birth defects in the country.

**Results: **Women who conceived when residing in Ruiru sub-county (adjusted odds ratio [aOR]: 5.28; 95% CI; 1.68-16.58; P<0.01), and Kiambu sub-county (aOR: 0.27; 95% CI; 0.076-0.95; P=0.04), and preceding siblings with history of birth defects (aOR: 7.65; 95% CI; 1.46-40.01; P=0.02) were identified as the significant predictors of major external structural birth defects in the county.

**Conclusions:** These findings pointed to MESBDs of genetic, multifactorial inheritance, and sociodemographic-environmental etiology. Thus, we recommend regional defect-specific surveillance programs, public health preventive measures, and treatment strategies to understand the epidemiology and economic burden of these defects in Kenya. We specifically recommend the integration of clinical genetic services with routine reproductive health services because of potential maternal genetic predisposition in the region.

## Introduction

Worldwide, an estimated 7.9 million children are born every year with a birth defect of which approximately 3.3 million die before age five and around 3.2 million could be physically disabled for life ^1, 2^. More than 94% of such defects occur in developing countries where about 95% of these children do not survive beyond childhood ^1^. Birth defects are defined as abnormalities of body structures or functions that develop during the organogenesis period (first trimester of gestation) and detectable during pregnancy, at birth, or soon after ^2, 3^. These defects may be classified as major when associated with significant adverse health effects requiring medical/surgical care; otherwise, they are described as minor ^1, 2^. Alternatively, they can be classified as external when visible at birth or soon after; or internal when advanced medical imaging techniques are required for their detection ^4– 6^. Consequently, the phrase ‘major external structural birth defects’ (MESBDs) denotes congenital physical abnormalities that are clinically obvious at birth or soon after which call for medical and/or surgical interventions ^1, 2^. The causes of these defects can be classified into three categories: (i) identifiable environmental factors (teratogens/micronutrient deficiencies); (ii) identifiable genetic factors; and (iii) complex genetic and idiopathic environmental factors, described as multifactorial inheritance ^1, 4, 7– 10^. One-third of these causes are attributed to identifiable environmental and genetic factors, whereas the rest are believed to be of multifactorial aetiology ^1, 4, 7– 10^. Additionally, an environmental endowment of women of reproductive age is thought to operate through their socioeconomic and sociodemographic characteristics leading to causes of MESBDs, described as sociodemographic-environmental factors ^1, 4, 8– 10^.

Organogenesis occurs in the first eight weeks of gestation; however, approximately half of pregnancies are usually unplanned/unintended, thus not recognized until the end of the second trimester ^1, 4, 11– 13^. Completing more years of education could improve maternal health because educated women are more likely to make informed reproductive health choices than those with low levels of education to improve birth outcomes ^14– 17^. Some of the notable maternal decisions include planned pregnancy, preconception folic acid intake in anticipation of conception, and subsequently prompt prenatal care ^14, 16, 18– 23^. Supplemental vitamins with folic acid are dispensed during routine antenatal care (ANC) visits, as well as health education on adequate nutrition, avoidance of environmental teratogens, and maternal infections as public health preventive strategies for MESBDs ^10, 24^. These measures could be effective only when pregnant women promptly began antenatal care within eight weeks of gestation before the intrauterine formation of MESBDs ^4^. Folic acid is essential for normal development of the brain and spinal cord during the first 4 weeks of conception, and have been found to reduce the occurrence of neural tube defects, orofacial clefts, limb reduction defects, urinary system defects, and omphalocele; some of the most prevalent defects in the county ^25– 27^. Thus, the recommended first ANC at the 12 ^th^ week of pregnancy could be a sub-optimal preventive strategy for these defects, nevertheless it improves experiences of the women during pregnancy and childbirth ^28^. Maternal occupation as a predictor of MESBDs could be dependent on educational levels, nonetheless occupations such as farming could expose women of reproductive age to teratogenic pesticides ^29^.

Maternal residence at conception is similarly a significant predictor of MESBDs determined by environmental etiology attributed to widespread poverty, environmental pollution, inadequate health care services, and ineffective preventive strategies; factors largely found in developing countries ^1, 7, 30^. Parental age is a multifaceted risk factor whose mechanisms of actions in the intrauterine formation of MESBDs are underpinned by human biology and socio-economic endowment among women of reproductive age. From the biologic standpoint, the female gametogenesis begins before birth with the initial meiotic division (prophase stage) expected to complete shortly before ovulation, however, this is not the case always because the process may delay up to 45 years to conclude ^24^. Thus, the oocytes take exceedingly long in the prophase stage increasing the likelihood of meiotic errors due to exposure to the environmental teratogens ^24^. Advancing maternal age beyond 35 years is similarly a risk factor for MESBDs of genetic etiology due to chromosomal abnormalities ^24, 31, 32^. Similarly, from the genetic viewpoint, genetic mutations and accumulation of chromosomal aberrations during the maturation of male germ cells have been attributed to the formation of MESBDs in utero ^33, 34^. The amount of deoxyribonucleic acid damage in sperm of men aged 36–57 is three times that of men <35 years, increasing the likelihood of these defects in aging couples ^34, 35^. From the socioeconomic perspective, parental age could be associated with MESBDs of multifactorial etiology ascribed to physiological interactions between complex genetic and idiopathic environmental attributes of women of reproductive age ^1, 7, 30, 36, 37^.

To our knowledge, many studies on the risk factors have been published in developed countries, however, such publications are scanty in developing countries owing to the rarity of the defects, unplanned/unintended pregnancies, and difficulties in identifying these women until the end of the second trimester when the defects have already formed ^4^. To address this gap, this study investigated maternal periconceptional exposure to environmental teratogens, sociodemographic-environmental, and multifactorial risk factors for MESBDs in Kiambu County, Kenya. The study assessed: maternal periconceptional exposure to farm-sprayed pesticides, and teratogenic therapeutic medicines proxied by maternal chronic illnesses (epilepsy and depression); multifactorial inheritance proxied by the history of siblings with birth defects, sex of the “last born” current child, nature of pregnancy, and parity; and sociodemographic-environmental factors consisting of maternal age, paternal age, residence, level of education, occupation, and adequate prenatal care proxied by gestational age at first ANC, and preconception folic acid intake. The findings of this study could provide great public health opportunities for the formulation of specific treatment strategies, preventive measures, risk-based surveillance systems, and clinical genetic services for the most prevalent MESBDs, regionally and nationally. Consequently, the objective of this study was to identify the risk factors for MESBDs among children in Kiambu County, Kenya.

## Methods

### Study design and settings

A hospital-based case-control study was conducted to identify the risk factors for MESBDs. The study participants were recruited as they presented to the child welfare clinics, neonatal/pediatric units, and occupational clinics for care during the data collection period from May 31 ^st^ 2019 to July 31 ^st^ 2019. A case-control design was the optimal design for this study considering its suitability for the investigation of rare outcomes, as is the case with MESBDs. Even though a population-based design would have been preferable, the ease of recruiting case and control subjects within the hospital settings disproportionately favored the hospital-based design. This was an observational study, therefore was reported as per the STROBE guidelines ^38^.

The study was conducted in 13 hospitals comprising three-county referral hospitals (Kiambu, Gatundu, and Thika), eight sub-county hospitals (Karuri, Kihara, Wangige, Nyathuna, Lari, Tigoni, Lussigetti, and Kigumo), and two faith-based hospitals (Presbyterian Church of East Africa Kikuyu Orthopedic and African Inland Church Cure International) situated within Kiambu County, Kenya. Notably, neither population-based nor hospital-based surveillance systems for MESBDs existed in the county nor the study hospitals. Nonetheless, cases detected by primary health providers during childbirth and neonatal care were recorded for the compilation of monthly hospital reports and subsequent entry into the District Health Information System (DHIS). The cases were drawn from Kiambu, Thika, Gatundu, Tigoni, Kikuyu, and Cure hospitals, which provided occupational and rehabilitative health services to children with MESBDs. The controls, on the other hand, were drawn from Kiambu, Gatundu, Thika, Karuri, Kihara, Wangige, Nyathuna, Lari-Rukuma, Tigoni, Lussigetti, and Kigumo hospitals which provided child welfare services to the under-fives. Kiambu is the second-most densely inhabited county with an estimated population of 2.4 million people out of an estimated national population of 47.5 million ^39^. Its economic mainstay is largely agriculture, comprising tea, coffee, and dairy farming ^39^. Of the county’s total estimated population, approximately 2.2% aged ≥5 years are living with lifelong disabilities ^39^. A study carried out in the county between 2014 and 2018 observed defects of the musculoskeletal system as the most prevalent single system defects followed by central nervous, orofacial clefts genital, ocular, and anal organ defects ^25^.

### Study population and eligibility of participants

The study population consisted of children aged ≤5 years old seeking health services at the study hospitals during the study period spanning from May to July 2019. All children whose mothers consented to participate in the study were recruited.

### Case definition and recruitment

Cases were defined as children aged ≤5 years born with at least one MESBD to resident women of Kiambu County and seeking health care services at the neonatal units, pediatric wards, child welfare clinics, and/or occupational therapist clinics of the study hospitals during the three-month study period. The Research Assistants (RAs) liaised with team leads of the departments listed above to identify cases of MESBDs. The team leads had been working in these departments, thus were conversant with the cases seeking services. The team leads invited the mothers of the children who met the case definition to comfortable private rooms within the departments where informed consent was sought and interviews conducted by the RAs. All cases that met this definition and whose caregivers consented to participate were prospectively recruited into the study until the required sample was attained (see *Sample size determination*).

### Control definition and recruitment

Controls were defined as children aged ≤5 years born without any forms of birth defects to resident women of Kiambu County and attending routine child welfare clinics at the study hospitals during the same three-month study period. The Research Assistants liaised with team leads of the child welfare clinics to identify the children without any form of birth defects and were seeking routine immunization, and growth monitoring services. The team leads had been working in these clinics, hence were familiar with most of the under-fives seeking the services. These services are provided between 8.00 am and 5.00 pm from Monday to Friday; the team leads introduced the RAs who then briefed the potential participants on the study objectives. Because of the relatively large number of controls available, they were selected by simple randomization using sealed envelopes upon definition of the sample population and frequency-matched to the cases by the day of presentation. Informed consent was sought from the study participants who met the study eligibility criteria; those who consented to participate in the study were prospectively recruited and invited to secluded comfortable rooms within the clinics where face-to-face interviewer questionnaires were administered till the desired sample size was achieved ( ***see sample size determination***). 

### Sample size determination

The sample size was estimated as per the Kelsey JL *et al.* ^40^ ** formula specified for case-control studies as follows: -

$$n_{1}=\frac{{\left(Z_{\alpha }+Z_{\beta }\right)}^{2}\bar{pq}(r+1)}{r{\left(p_{1}-p_{2}\right)}^{2}}$$

$$\bar{q}=1-\bar{p}$$

$$n_{2}=rn_{1}$$

$$p_{1}=\frac{p_{2}OR}{1+p_{2}(OR-1)}$$

$$\bar{\;p}=\frac{p_{1}+rp_{2}}{r+1}$$

Where: *n*_1_ is the number of cases and *n*_2_ is the number of controls; *p*_1_ is the proportion of cases whose caregivers did not begin prenatal care in the first trimester (primary exposure), *p*_2_ is the proportion of controls whose caregivers did not begin prenatal care in the first-trimester set at 57% ^11, 12^. Remarkably, *Z _α_*_/2_ (1.96) and *Z _β_* (-0.84) are the values specifying the desired two-tailed confidence level (95%) and statistical power (80%), respectively. The odds ratio *(OR)* for the effect of the primary exposure (cases whose caregivers did not begin prenatal care in the first trimester) was hypothesized to be 2.0 (universally accepted). The ratio *(r)* of unexposed to exposed individuals was set at 3.0, and given the estimates, a total sample size of 408 participants was derived (102 cases, and 306 controls).

### Data collection process and study variables

Before data collection, four nursing graduate interns were recruited and trained as RAs on sound interviewing techniques, and information derivation/validation from antenatal care (ANC) booklets. This was to ensure the data collection process spanning three months (May 31 ^st^ 2019 to July 31 ^st^, 2019) was conducted in a standardized manner. The ANC booklet contains maternal profile, medical/surgical history, previous pregnancy history, clinical notes, and physical examination findings on ANC visits, among others. The maternal profile includes name, age, parity gravidity, height, weight, last menstrual period (LMP), expected date of delivery (EDD), and date of first ANC. Face-to-face structured questionnaires (see *Extended data*) were administered to the mothers of the study participants by RAs in comfortable secluded rooms within neonatal units and occupational therapy clinics for cases and child welfare clinics for the controls. Data were gathered retrospectively on exposures to environment-teratogens (farm-sprayed pesticides, and teratogenic medicines proxied by chronic illnesses), multifactorial inheritance (parity, nature of pregnancy, history of siblings with birth defects and sex of the “lastborn” (current) child), and sociodemographic-environmental factors (maternal age, paternal age, residence, education level, occupation, and adequate prenatal care proxied by gestational age at first ANC and preconception folic acid intake). The predictors were assessed as shown in Table 1.

**Table 1.** Study variables and their assessments.

Variable (type)	Method of assessment
Exposure to farm-sprayed pesticides (nominal)	Captured as “yes” for those who sprayed farms with pesticides and “no” for those who did not spray farms with pesticides
Teratogenic therapeutic medicines for chronic illnesses (nominal)	Captured as a nominal variable, categorized, and labelled; 1= “medicines for hypertension”, 2= “no medicines for chronic illnesses” and 3= “medicines for other chronic illnesses”
ANC began 8 weeks post-conception began (nominal)	Captured as yes/no
Gestational age (weeks) at first ANC (continuous)	Captured in weeks, categorized, and labelled; 1<9 weeks, and 2≥ 9 weeks at first ANC visit.
Preconception folic acid intake (nominal)	Captured as yes/no
Sex of the “lastborn” current child (nominal)	Entered as male or female
History of siblings with birth defects (nominal)	This was captured as yes/no
Parity (continuous)	Abstracted from the ANC booklet as a continuous variable, categorized as and labelled; =1= “primiparous”, and >1= “multiparous”
Nature of pregnancy (nominal)	Entered as single or multiple
Maternal age (continuous)	Captured in years
Paternal age (continuous)	Captured in years
Level of education (ordinal)	Captured as no schooling, primary, secondary, college certificate, college diploma, and university degree, categorized and labelled; 1≤ primary, 2=secondary, and 3=tertiary
Maternal occupation (nominal)	Captured as a nominal variable, categorized into three groups: 1=farming, 2=employed, and 3=unemployed.
Residence (nominal)	Captured as a nominal variable, and categorized into five groups: 1=Thika, 2=Gatundu, 3=Kiambu, 4=Ruiru, and 5=other sub-counties

ANC, antenatal care; MESBDs major external structural birth defects.

**Figure 1.**
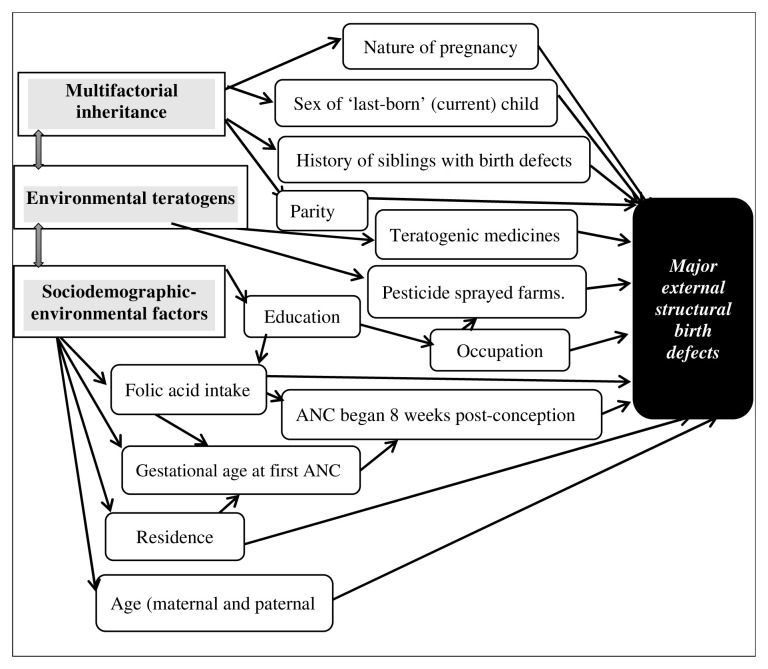
Causal diagram of factors thought to influence major external structural birth defects (MESBDs) among children in Kiambu County, Kenya.

**Figure 2.**
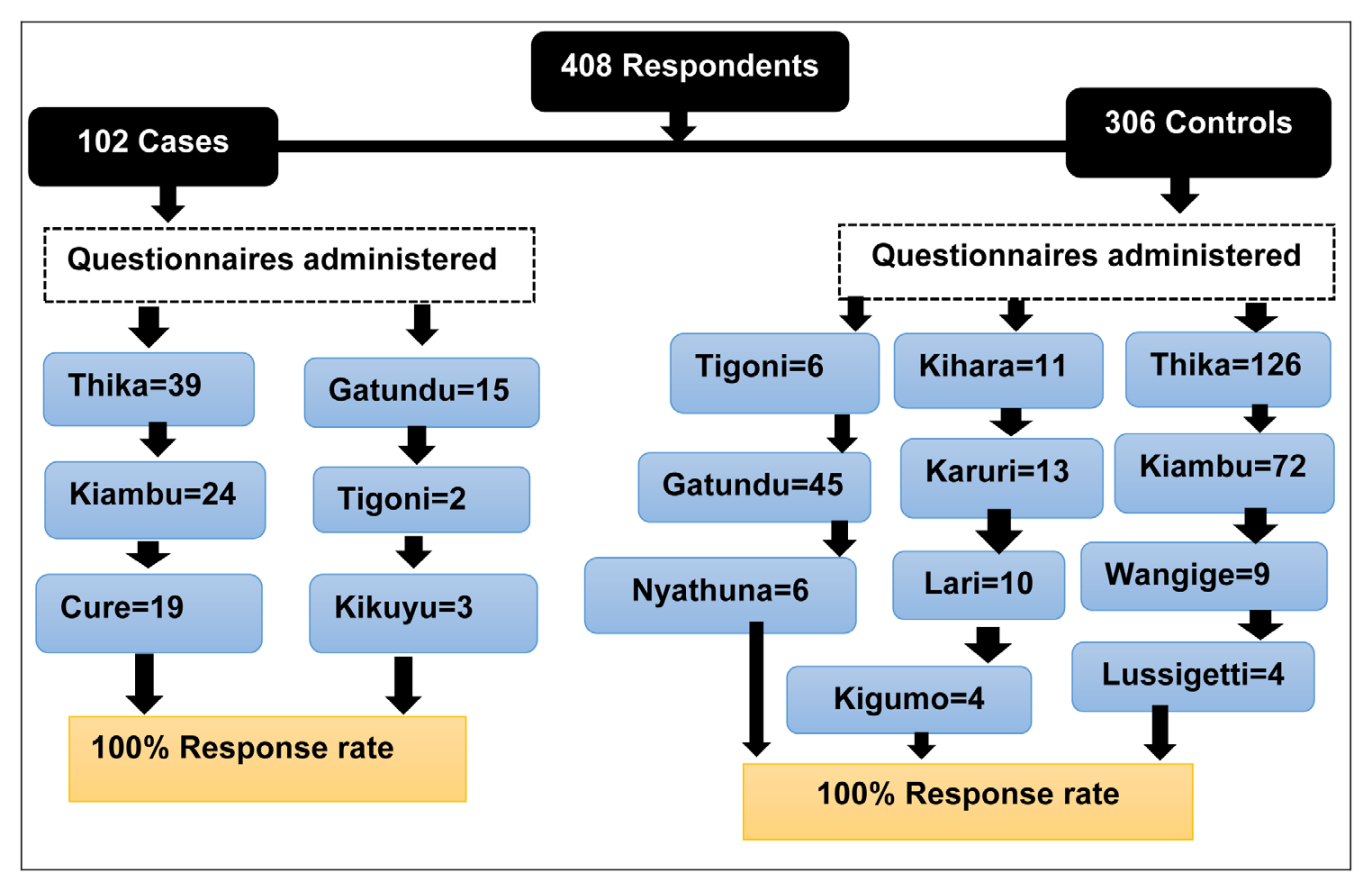
Flow chart of the systematic sampling strategy used in this study.

The conceptual framework was organized based on the three causal categories of MESBDs (multifactorial inheritance, environmental teratogens, and sociodemographic-environmental factors). Nonetheless, because disentangling genetic etiology (identifiable, and complex) was a scientific limitation of observational studies as is the case in our study, analysis of such factors sufficed as a multifactorial inheritance in this conceptual framework to measure maternal genetic predispositions. A conceptual framework depicting the predictor-outcome relationship is displayed in Figure 1. The flow chart of the simple-random systematic sampling strategy is shown in Figure 2.

### Ethical considerations

Ethical approval for this study was obtained from the Kenyatta National Hospital [KNH]-University of Nairobi [UoN] Ethics Review Committee [Ref. No: KNH-ERC/A/44]. The purpose of the study was explained to participants and written informed consent was obtained from the mothers of the study subjects before engaging in the study.

### Minimizing bias

Considering potential biases inherent in case-control studies that were likely to invalidate the study results, deliberate attempts were made to minimize their occurrence. First and foremost, the research assistants were trained on sound interviewing techniques and information derivation/validation from ANC booklets to minimize interviewer and minimize information biases, respectively. In a bid to minimize recall bias, gestational age at the first ANC was estimated from the dates of the last menstrual period and dates of the first ANC obtained from the ANC booklets.

### Data processing and statistical analysis

Following data collection, filled questionnaires were manually checked daily for accuracy and completeness and subsequently entered into a Microsoft Excel spreadsheet (Microsoft Office Professional Plus 2019) by two independent data managers to reduce potential errors. The excel dataset was validated and exported to Stata software version 14.0 (Stata Corporation, Texas, USA) for further cleaning, coding, and analyses. Descriptive analyses (means, medians, standard deviations, and ranges) were used to summarize continuous variables, whereas proportions and percentages for categorical variables were generated and presented in frequency tables. Afterward, the effect of each predictor on the odds of MESBDs was assessed using univariable logistic regression models at a liberal P-value (P≤0.20) ^41^. Gestational age at first ANC as a continuous variable was categorized into groups (<9 weeks and ≥9 weeks) for evaluation in the univariable analyses ^1, 4, 11– 13^. Additionally, parity as a continuous variable was grouped into two groups: =1=primiparous or >1=multiparous categories for assessment in the univariable analyses ^42, 43^. However, maternal age as a continuous variable was insignificant in the univariable analyses, thus, recategorized into two groups; <35 years, and ≥35 and reassessed for statistical significance; women aged at least 35 years have previously been reported to have an increased likelihood of giving birth to children with MESBDs ^44^. Paternal age as a continuous variable was similarly insignificant in the univariable analyses, thus recategorized into seven groups and reassessed for statistical significance which was still insignificant. Nevertheless, paternal age was further recategorized into two groups (<35 years, and ≥35) and reassessed for statistical significance yet still insignificant; males aged at least 35 years have previously been associated with increased likelihood of defect-affected births in their female counterparts ^34^. Variables found statistically significant in the univariable analyses were fitted to a multivariable model where a backward stepwise approach was used to eliminate variables from the model at P-value >0.05. Nature of pregnancy was however collinear in the multivariable analyses thus dropped in the final multivariable analysis. To minimize the confounding effects, elimination of non-significant predictors was only considered when their exclusion from the model did not yield more than a 30% change in the effects of the remaining variable ^41^. Two-way interactions were fitted between the remaining variables of the final model and assessed for significance. A Hosmer-Lemeshow test was used to assess the goodness of fit of the logistic model, with a P-value of >0.05 being suggestive of a good fit.

## Results

A total of 408 study respondents (102 cases and 306 controls) were enrolled in this study. The cases consisted of cleft lip with palate 1 (0.98%), cleft palate 3 (9.94%), clubbed hand 1 (0.98%), club foot 91 (89.22%), hydrocephalus 1 (0.98%), limb defects 4 (3.92%), and persistent cloacal 1 (0.98%) ^45^.

### Descriptive statistics

*Sociodemographic-environmental factors:* The median age of the study respondents was 26 years with a mean of 27.31 years (SD=5.73, R; 17-47) ( Table 2). The median age of mothers in the case group was 28 years with a mean of 28.73 (SD=5.95, R; 19-47), whereas the median age of mothers in the control group was 26 years with a mean of 26.84 (SD=5.58, R; 17-42) ( Table 2). The mean paternal age of the study respondents was 32.02 years with a standard deviation of 6.34 years, and a median age of 31 years ranging between 19 and 56 years ( Table 2). Of the 408 study participants, 184 (45.10%) had attained a secondary level of education; 38 (37.25%) and 146 (47.71%) in the case and control groups, respectively ( Table 2).

*Environmental-teratogens:* Of the 408 study respondents, 15 (3.68%) were exposed to farm-sprayed pesticides, of which four (3.92%) were in the case group and 11 (3.59%) were in the control group ( Table 2).

**Table 2.** Descriptive statistics of the study respondents (N=408).

Variables	Measurements	Observations (N=408), n (%)	Cases (N=102), n (%)	Controls (N=306), n (%)
Residence	Thika	125 (30.64)	33 (32.35(	92 (30.07)
	Gatundu	62 (15.20)	13 (12.75)	49 (16.01)
	Kiambu	104 (25.49)	15 (14.71)	89 (29.08)
	Ruiru	38 (9.31)	20 (19.61)	18 (5.88)
	Others	79 (19.36)	21 (20.59)	58 (18.95)
Maternal age	<35	356 (87.25)	82 (80.39)	274 (89.54)
	≥35	52 (12.75)	20 (19.61)	32 (10.46)
Mean		27.31	28.73	26.84
Median		26	28	26
Standard deviation (SD)		5.73	5.95	5.58
Range (R)		17-47	19-47	17-42
Paternal age	<35	251 (67.11)	64 (70.33)	187 (66.08)
	≥35	123 (32.29)	27 (29.67)	96 (33.92)
Mean		32.02	31.3	32.25
Median		31	30	31
Standard deviation (SD)		6.34	5.47	6.59
Range		19.56	21-54	19-56
Maternal education	≤Primary	94 (23.04)	27 (26.47)	67 (21.90)
	Secondary	184 (45.10)	38 (37.25)	146 (47.71)
	Tertiary	130 (31.86)	37 (36.27)	93 (30.39)
Maternal occupation	Farming	24 (5.88)	7 (6.86)	17 (5.56)
	Unemployed	206 (50.49)	40 (39.22)	166 (54.25)
	Employed	178 (43.63)	55 (53.92)	123 (40.20)
Parity	Primiparous	127 (37.35)	28 (35.00)	99 (38.08)
	Multiparous	213 (62.65)	52 (65.00)	161 (61.92)
Mean		2.12	2.14	2.12
Median		2	2	2
Standard deviation (SD)		1.21	1.41	1.22
Range (R)		1–8	1–6	1–8
Nature of pregnancy	Multiple	5 (1.23)	3 (2.94)	2 (0.65)
	Single	403 (98.77)	99 (97.06)	304 (99.35)
Sex of the “lastborn” current child	Female	199 (48.77)	45 (44.12)	154 (50.33)
	Male	209 (51.23)	57 (55.88)	152 (49.67)
Sibling with a history of birth defects	No	393 (96.32)	93 (91.18)	300 (98.04)
	Yes	15 (3.68)	9 (8.82)	6 (1.96)
Gestational age at first ANC	<9 weeks	23 (9.09)	9 (18.75)	14 (6.83)
	≥9 weeks	230 (90.91)	39 (81.25)	191 (93.17)
Mean		20.1	18.35	20.40
Median		20	18	21
Standard deviation (SD)		7.54	8.13	7.36
Range		4–40	4–35	4–40
Exposure to farm-sprayed pesticides	No	393 (96.32)	98 (96.08)	295 (96.41)
	Yes	15 (3.68)	4 (3.92)	11 (3.59)
Teratogenic therapeutic medicines for chronic illnesses	Medicines for hypertension	17 (4.17)	4 (3.92)	13 (4.25)
	No medicines for chronic illnesses	382 (93.63)	96 (94.12)	286 (93.46)
	Medicines for others chronic illnesses	9 (2.21)	2 (1.96)	7 (2.29)
Preconception folic acid intake	No	230 (56.65)	59 (57.84)	171 (56.25)
	Yes	176 (43.35)	43 (42.16)	133 (43.75)
ANC began eight weeks post- conception	No	330 (80.88)	77 (75.49)	253 (82.68)
	Yes	78 (19.12)	25 (24.51)	53 (17.32)

SD, standard deviation; R, range; Gatundu North and South sub-counties categorized as Gatundu sub-county, whereas Thika East and West sub-counties categorized as Thika sub-county.

*Multifactorial inheritance:* Of the 408 study respondents, 404 (98.77%) had single gestations for the current child, of which 99 (97.06%) and 304 (99.35%) were in the case and control groups, respectively ( Table 2). Of the study participants, approximately 3.68% (15) of the study participants reported a history of siblings with birth defects consisting of 9 (8.82) in the case group and 6 (1.96) in the control group ( Table 2). Of the 15 study participants, 12 stated the name or described the nature of the defects in their previous pregnancies/births, however, 3 participants were unable to do so. Of the 12 study respondents, 7 of the case subjects with congenital talipes equinovarus reported a history of birth defects in their previous births of which 4 subjects reported a recurrence of congenital talipes equinovarus, whereas 3 reported foot aversion, internally rotated shorthand (phocomelia), and congenital scoliosis. On the other hand, 5 control subjects reported a history of siblings with birth defects in their preceding births comprising 3 cases of congenital talipes equinovarus, 1 case of autism, and 1 case of deafness ( Table 3).

**Table 3.** History of siblings with birth defects among case and control subjects.

Types of MESBDs	Cases (n=102)	Controls (n=306)	Total (n=408)
Congenital talipes equinovarus	4	3	7
Autism		1	1
Deafness		1	1
Foot aversion	1		1
Internally rotated shorthand	1		1
Congenital scoliosis	1		1
**Total**	**7**	**5**	**12**

### Logistic regression analyses

Notably, the factors assessed for statistical significance in the univariable analyses and found associated with MESBDs at P≤0.20 included maternal age, residence, education, occupation, ANC visits beginning eight weeks post-conception, gestational (age) at first ANC visits, nature of pregnancy, and history of siblings with birth defects ( Table 4). Subsequently, these variables were fitted to the multivariable model for the final analysis, except education being distal relative to occupation, gestational age at first ANC visits, and ANC beginning eight weeks post-conception. ( Figure 1).

**Table 4.** Univariable analysis of factors associated with MESBDs among children in Kiambu County, Kenya.

Variable	Value	Odds ratio	95% CI	P-value
Residence*	Others	Reference		
	Thika	0.99	0.52-1.86	
	Gatundu	0.73	0.33-1.61	<0.001
	Kiambu	0.47	0.22-0.98	
	Ruiru	3.07	1.37-6.89	
Maternal age *	<35	Reference		
	≥35	2.09	1.13-3.85	0.02
Paternal age	≥35	Reference		
	<35	1.22	0.73-2.03	0.45
Maternal education *	Tertiary	Reference		
	Secondary	0.65	0.39-1.10	0.18
	≤Primary	1.01	0.56-1.82	
Maternal occupation *	Farming	Reference		
	Employed	1.09	0.43-2.77	003
	Unemployed	0.59	0.23.1.51	
Preconception folic acid intake	No	Reference		
	Yes	0.94	0.60-1.47	0.78
ANC began eight weeks post gestation *	No	Reference		
	Yes	1.55	0.90-2.66	0.11
Gestational age at first ANC *	<9 weeks	Reference		
	≥9 weeks	0.32	0.13-0.79	0.01
Parity	Primiparous	Reference		
	Multiparous	1.14	0.68-1.93	0.62
Nature of pregnancy *	Multiple	Reference		
	Single	0.22	0.04-1.32	0.10
Sex of the “lastborn” current child	Female	Reference		
	Male	1.28	0.82-2.01	0.28
Siblings with a history of birth defects *	No	Reference		
	Yes	4.84	1.68-13.95	<0.01
Teratogenic therapeutic medicines for chronic illnesses	No medicines for chronic illnesses	Reference		
	Medicines for hypertension	0.92	0.29-2.88	1.0
	Medicines for other chronic illnesses	0.85	0.17-4.17	
Exposure to farm-sprayed pesticides	No	Reference		
	Yes	1.09	0.34-3.52	0.88

*Variables eligible for inclusion in the multivariable model (P≤0.20). CI, confidence interval; MESBD, a major external structural birth defect.

In the multivariable analysis, only maternal residence at conception, and history of siblings with birth defects were shown as the significant predictors MESBDs at a 5% significance level ( Table 5). Compared to women who conceived while residing in other sub-counties, women who conceived when residing in Ruiru sub-county were 5.28 times likely to give birth to children with MESBDs (aOR: 5.28; 95% CI: 1.68-16.58; P<0.01); whereas women who conceived when residing in Kiambu sub-county were 27% less likely give birth to children with MESBDs (aOR: 0.27; 95% CI; 0.076-0.95; P =0.04) holding all factors constant. Additionally, compared to siblings without a history of birth defects, siblings with a history of birth defects were 7.65 times likely to be born with MESBDs (aOR: 7.65; 95% CI; 1.46-40.01; P =0.02) holding all factors constant ( Table 5).

**Table 5.** Multivariable analysis of factors associated with MESBDs among children in Kiambu County, Kenya.

Variable	Value	aOR	95% CI	P-value
Maternal residence	Other sub-counties	Reference		
	Kiambu	0.27	0.076-0.95	0.04
	Ruiru	5.28	1.68-16.58	<0.01
Siblings with a history of birth defects	No	Reference		
	Yes	7.65	1.46-40.01	0.02

aOR, adjusted odds ratio; CI, confidence interval; MESBD, a major external structural birth defect.

## Discussion

To our knowledge, this was the first case-control study conducted to identify the risk factors for MESBDs in the entire county. Our study results mimicked other findings across the world that maternal residence at conception and history of siblings with birth defects are strongly associated with the intrauterine formation of MESBDs ^1, 30, 46^. Our study observed orofacial clefts comprising 1 (0.98%) cleft lip with the palate, and 3 (9.94%) cleft palates; limb reduction defects comprising 1 (0.98%) clubbed hand, and 4 (3.92%) limb defects; defects of the musculoskeletal system consisting of 91 (89.22%) clubfeet; and neural tube defects comprising 1 (0.98%) hydrocephalus and 1 (0.98%) persistent cloacal. These are some types of MESBDs associated with genetic, partially genetic, and multifactorial etiology ^1, 30, 46^. The prevalence of such defects have been observed to vary by regions attributed to ethnical, and socioeconomic differences globally ^1, 30^. Siblings with a positive history of MESBDs among their preceding siblings are at most risks of being born with MESBDs, have a recurrence of similar defects among the siblings, and/or among their offspring ^46^. This was indeed evident in this study where 4 of the case subjects with clubfoot similarly reported clubfoot in their preceding siblings, whereas 3 of the case subjects with clubfoot reported foot aversion, internally rotated shorthand (phocomelia), and congenital scoliosis each in their preceding siblings. Our study similarly made remarkable observations where case subjects with clubfoot reported concurrence of congenital pes planus, and arthrogryposis each, whereas a case subject with hydrocephalus reported concurrence of congenital pes planus, and two case subjects of limb defects reported concurrence with Down syndrome each. On the other hand, 5 control subjects reported a history of siblings with birth defects in the preceding births comprising 3 cases of clubfoot, 1 case of autism, and 1case of deafness.

Positive siblings and familial history of specific types of MESBDs have been associated with increased risks of recurrence in subsequent pregnancies ^24, 46, 47^. Worldwide, the recurrence rate of NTD and Down syndrome have been approximated at 2-5% and 1%, respectively ^24, 46, 47^. Thus, accurate knowledge of birth defects by families when given to the clinicians is similarly of public health significance to improve risk assessments and reproductive health planning for couples susceptible to birth defects of genetic, and multifactorial origin ^46^. Even though our study did not show a significant statistical association between MESBDs with parental age, advanced age has been strongly associated with defects of chromosomal etiology (Down syndrome), and non-syndromic etiology (neural tube defects and orofacial clefts) ^1, 30, 34, 48,^. Nonetheless, our study alluded to an increased risk of chromosomal abnormalities thus suggestive of the prevalence of MESBDs of genetic origin in the county. High prevalence of Down syndrome has been observed in developing countries attributed to many older women becoming pregnant, limited family planning services, unavailability of prenatal genetic screening, diagnosis, and related services ^1, 30^. MESBDs are considered defects of public health importance, however the presence of certain defects; rare or common, minor or major, internal or external, functional or structural sometimes act as pointers to latent defects of similar significance because of the multiple genetic epidemiology, thus diagnosable later using advanced medical imaging techniques ^3, 6, 46^.

Our study similarly observed maternal residence at conception as a predictor of the intrauterine formation of MESBDs. The study showed that women who got pregnant when residing in Ruiru sub-county were 5.28 times likely to give birth to children with MESBDs compared to those who got pregnant residing in other sub-counties within Kiambu County. Conversely, the study showed that women who got pregnant when residing in Kiambu sub-county were 27% less likely to give birth to children with MESBDs compared to those who got pregnant residing in other sub-counties within the county. The study showed that Kiambu sub-county was protective implying it was relatively safe for women of reproductive age to become pregnant while residing in the sub-county. Maternal residence at the time of conception as a risk factor for MESBDs could be ascribed to variations in maternal genetic, multifactorial, sociodemographic-environmental attributes. From the genetic perspective, increased frequency of single-gene defects in developing countries has been associated with increased frequency of common recessive disorders such as hemoglobin disorders, sickle cell anemia, thalassemia, oculocutaneous albinism, and cystic fibrosis because of the discerning advantage for carriers to the mortal effects of malaria, as well as recessive conditions associated with high rates of consanguineous (cousin) marriages ^1, 30^. Additionally, high prevalence of defects of chromosomal etiology in developing countries have been ascribed to women delaying childbearing beyond 35 years, limited maternal access to family planning services, and absence of clinical genetic services ^1, 24, 30, 48^. Sociodemographic-environmental characteristics, and physiological interactions between complex genetic disorders, and idiopathic environmental factors could also lead to the occurrence of MESBDs associated with ethnic and geographic differences ^1, 30^. Thus, the epidemiology of MESBDs in the county underscore an underlying genetic, multifactorial, sociodemographic-environmental etiology contributing to the global debate on the burden of a “silent” public health problem in developing countries ^1, 30^.

Although our study did not show an association between MESBDs with known environmental factors (teratogens and micronutrient deficiencies), pregnancies in developing countries are at increased risk of potential teratogens because of high prevalence of intrauterine infections, maternal malnutrition, low socioeconomic levels, low levels of education, deficient environmental protection policies, and insufficiently regulated access to medicines ^1, 30^. This could imply the county is performing relatively well in controlling potential environmental causes of MESBDs. The teratogens consist of; (i) congenital infections; (ii) maternal and altered metabolism; and (iii) recreational and therapeutic drugs ^1, 30^. Congenital infections comprise toxoplasmosis, other infections (syphilis, varicella-zoster, human parvovirus B19), rubella, cytomegalovirus, and herpes, denoted by an acronym “TORCH” ^1, 30^. Epilepsy and insulin-dependent diabetes are the examples of maternal illnesses and altered metabolism, whereas statins and alcohol are the examples of therapeutic and recreational drugs, respectively ^1, 30^. Our study also did not show significant associations between MESBDs with maternal occupation, gestational age at first ANC, and ANC beginning 8 weeks post-conception; factors thought to influence maternal iron-folic acid supplementation ^14, 16, 21^. Folic acid is crucial for the biosynthesis, and methylation of deoxyribonucleic acid (DNA) and ribonucleic acid (RNA) which are important for cell division, differentiation, and regulation of gene expression, during rapid cell division such as embryogenesis, thus is necessary for the growth and smooth functions of human cells ^24, 49^.

Nevertheless, some limitations were inherent in this study; there was a likelihood of differential recall bias among the study respondents; cases were more likely to remember their preconception period owing to the experience of MESBDs in the last birth than the controls, thus recall bias could affect estimates of the odds ratios. The study participants with a history of siblings with birth defects either stated or described the nature of the defects however the researchers could not ascertain accuracy of the diagnoses/descriptions, while others did not know the names of the defects. Survivor bias was also an inherent limitation in this study because some defects such as neural tube defects are potentially fatal, however the study could not establish the causes of deaths among stillbirths, and miscarriages in the study hospitals because it was not a pathological standard operating procedure in the entire Kenya. Additionally, due to the extreme rarity and stochasticity of MESBDs because of the absence of public health surveillance systems, the researchers lumped all types of MESBDs in calculating the sample size, yet births defects are largely heterogenous in their etiology, thus could also lead to underestimation of the effects of the predictors on the odds of MESBDs.

## Conclusions

These findings were indeed suggestive of genetic, multifactorial, and sociodemographic-environmental etiology of MESBDs in Kiambu County, Kenya. Thus, these findings could provide the greatest public health opportunities for health planners in the region to establish defect-specific surveillance programs, implement proven public health preventive strategies, and provide appropriate treatment interventions for the most prevalent MESBDs. Therefore, we would like to provide the following priority public health policy recommendations; establishment hospital-based surveillance systems for the most common MESBDs, and integration of clinical genetic services with routine reproductive health services, nationally. The genetic services should consist of counseling, screening, diagnosis, and associated treatments including elective termination of pregnancies for anomalies in jurisdictions with favorable legislative frameworks. Additionally, we would recommend further epidemiological, and economic evaluation studies to understand the epidemiology and economic burden of these defects in Kenya.

## Data availability

### Underlying data

Havard Dataverse: Risk factors for major external structural birth defects among children in Kiambu County, Kenya: a case-control study. https://doi.org/10.7910/DVN/PYLVUW

### Extended data

Harvard Dataverse: Risk factors for major external structural birth defects among children in Kiambu County, Kenya: a case-control study. https://doi.org/10.7910/DVN/PYLVUW

This project contains the following extended data:

- **Questionnaire_**mesbds_kmbu_ke.pdf (copy of questionnaire)
- **Dofile**_mesbds_kmbu_ke.do (syntax used for analysis)

Data are available under the terms of the Creative Commons Zero "No rights reserved" data waiver (CC0 1.0 Public domain dedication).
