# Supplementary material for: Risk factors for major external structural birth defects among children in Kiambu County, Kenya: a case-control study
Source: F1000Res. 2021 Apr 30;10:59. Originally published 2021 Feb 1. [Version 2] doi: 10.12688/f1000research.50738.2 (PMC8039862; doi:10.12688/f1000research.50738.2)
Supplement: Peer review history

# Peer review: Reviewer response for version 2

**DOI:** 10.5256/f1000research.56198.r84270

**Published:** 2021-05-25

**Authors:** Rogath Kishimba

**Affiliations:** 1 Tanzania Field Epidemiology and Laboratory Training Program, Dar es Salaam, Tanzania; 2 Tanzania Ministry of Health, Dodoma, Tanzania

The manuscript has improved a lot however the below issues need to be addressed before indexed. 

**Title:** When you look on studied birth defects you realize they are all non fatal may be untreated hydrocephalus if any can be fatal. I advice they change the title to read, "Risk factors for **non fatal** major external structural birth defects among children in Kiambu County, Kenya: a case-control study".

**Abstract:** Well written

**Methods:** 

- There is no a definition of "resident women". This is very important as it may link environmental factors and the observed outcome. 
- Recall bias is not only subjected to gestation age at first ANC. There are a lot of questions which you asked referring to the index pregnancy such as teratogenic medicines, folic acid intake etc. and when exactly they used. How did you mitigate this? It needs clarification.
- What is your sampling unit? A mother? A Newborn? How did you take care of multi pregnancy? Suppose both or all offspring had a birth defect.

**Results: **Analysis of newborn age is important to be included under the results section.

Is the work clearly and accurately presented and does it cite the current literature?

Partly

If applicable, is the statistical analysis and its interpretation appropriate?

Yes

Are all the source data underlying the results available to ensure full reproducibility?

Partly

Is the study design appropriate and is the work technically sound?

Partly

Are the conclusions drawn adequately supported by the results?

Partly

Are sufficient details of methods and analysis provided to allow replication by others?

Partly

Reviewer Expertise:

Maternal and Child Health Epidemiologist

I confirm that I have read this submission and believe that I have an appropriate level of expertise to confirm that it is of an acceptable scientific standard, however I have significant reservations, as outlined above.

# Peer review: Reviewer response for version 2

**DOI:** 10.5256/f1000research.56198.r84272

**Published:** 2021-05-20

**Authors:** Marcia Feldkamp

**Affiliations:** 1 Department of Pediatrics, University of Utah, Salt Lake City, UT, USA

My only other comment is regarding table 4. The authors revised this table and deleted p values for the reference group which is appropriate. However, they also deleted p values for exposures that were not the reference group but were or were not statistically significant.

Is the work clearly and accurately presented and does it cite the current literature?

Yes

If applicable, is the statistical analysis and its interpretation appropriate?

Partly

Are all the source data underlying the results available to ensure full reproducibility?

Yes

Is the study design appropriate and is the work technically sound?

Yes

Are the conclusions drawn adequately supported by the results?

Partly

Are sufficient details of methods and analysis provided to allow replication by others?

Yes

Reviewer Expertise:

NA

I confirm that I have read this submission and believe that I have an appropriate level of expertise to confirm that it is of an acceptable scientific standard.

# Peer review: Reviewer response for version 1

**DOI:** 10.5256/f1000research.53822.r81820

**Published:** 2021-04-09

**Authors:** Rogath Kishimba

**Affiliations:** 1 Tanzania Field Epidemiology and Laboratory Training Program, Dar es Salaam, Tanzania; 2 Tanzania Ministry of Health, Dodoma, Tanzania

This is a good and important research area for newborn health particularly now when a lot has been done on infectious unlike non infectious diseases. We have observed a great decrease of infant mortality given the available maternal and newborn interventions on infectious diseases. A higher contribution of non infectious disease particularly birth defects may be observed on neonatal and infant mortality with time. Below are my inputs and comments regarding this study;

- The title, abstract and introduction are well written.
- Current citations were used. 
- The study design is appropriate however selection of cases was not appropriate given the study title and objectives. It can be admitted as one of study limitation. 
- Cases were sampled from child welfare clinics, neonatal/pediatric units, occupational and rehabilitation clinics. All these data sources represent survivors of  MESBDs and most probably non fatal MESBDs. It is difficult to get fatal MESBDs like neural tube defects (NTDs) cases from this subpopulation as majority will not survive to meet them in rehabilitation clinics. 
- The ascertainment period from the case definition is too high (5years and below). This may lead to potential recall bias as it will be very difficult for a mother to remember what happened in her pregnancy in the 3-4 years ago. Again may lead to recruitment of survivors and non fatal MESBDs cases. This could be mitigated for at least to consider/restrict enrolment into the study for children below 1 or 2 years only. 
- I understand well that the data sources were the above mentioned clinics which are complimented by the ANC booklets. However the methodology section again mentioned about DHIS and I was wondering whether it was also another data source which was used. It needs clarity for the reader to well understand sources of data for this study. 
- The methodology section need more clarity on maternal age. Is it the age of the mother during conception of the referred case? or the age of the mother during the data collection? It is also very important to define "residence" as it has implication on maternal exposures. The residence is important during conception and antenatal period.  This is the period when environmental exposures can have impact on the unborn child. There is no any significance of considering residence post delivery. 
- Sample size calculation is Ok. However you can not estimate proportion of controls ( *p*_2_) using a study with a different objectives from your intended study. 
- The hypothesized odds ratio for the effect of the primary exposure is too high. This is the risk which you allow to be detected in your study. At least you can allow a minimal risk of odds ratio between 1.5 and 2. 
- Results were well written however there is a need to your interpretation and conclusion to reflect your exact results. If the maternal age ≤34 years was found to be protective does not mean the maternal age ≥35 years is a risk. Remember this age category was your reference. If you want to refer the age category ≥35 years then make the other category " ≤34 years" a reference in your logistic regression analysis. Otherwise I advise to interpret and make conclusion exactly as what you found in your result section. 

Is the work clearly and accurately presented and does it cite the current literature?

Partly

If applicable, is the statistical analysis and its interpretation appropriate?

Yes

Are all the source data underlying the results available to ensure full reproducibility?

Partly

Is the study design appropriate and is the work technically sound?

Partly

Are the conclusions drawn adequately supported by the results?

Partly

Are sufficient details of methods and analysis provided to allow replication by others?

Partly

Reviewer Expertise:

Maternal and Child Health Epidemiologist

I confirm that I have read this submission and believe that I have an appropriate level of expertise to confirm that it is of an acceptable scientific standard, however I have significant reservations, as outlined above.

# Response

**Published:** 2021-04-11

**Authors:** George Agot

**Affiliations:** University of Nairobi, College of Health Sciences, School of Public Health, Kenya

**Sampling of cases: **Survivor bias has since been included as a limitation of this study  because some of these defects for example neural tube defects are potentially fatal, yet such data could not be gathered because we adopted a retrospective approach to gather the information.

**The ascertain period for cases:** It is true this period could potentially attract recall bias. This period was preferred considering the defects are extremely rare coupled with unavailability of surveillance systems in the county. Even though we preferred <5 years, we encountered children aged <4 years and approximately 78% were aged  <2 years, thus, this limitation was somehow reduced by default. 

**DHIS: **This was not used as a source of data in this study, however was intended to inform the readers on how data on birth defects are routinely gathered in the county in the absence of surveillance systems.

**Maternal age:** Maternal age was defined as maternal age at conception. 

**Residence:** Residence was defined as maternal place of residence at conception, thus introduced in the model and controlled for in multivariable logistic analysis. 

**Sample size calculation:**  The choice of literature for p2 was informed by maternal peri-conceptional period, especially 8 weeks of gestation when defects are expected to have formed yet most women do not plan their pregnancies, thus making it difficult for health care workers to identify these women in good time to effectively implement public health preventive measures before the defects form. Majority of such women attend first ANC at the end of second trimester of gestation when the defects have already formed.

**Hypothesized odds ratio:** Hypothesized odds ratio for the effects of the primary exposure is actually 2.0 (universally accepted) and not 3.0 as presented in the manuscript. This was a tying error and has been corrected. 

**Results:**  The reference category for maternal age was changed to <35 years, but showed no association in the univariable analysis.

**Conclusions:** The conclusions have been aligned to the study findings and interpretations.

# Peer review: Reviewer response for version 1

**DOI:** 10.5256/f1000research.53822.r81531

**Published:** 2021-04-07

**Authors:** Marcia Feldkamp

**Affiliations:** 1 Department of Pediatrics, University of Utah, Salt Lake City, UT, USA

The investigators present a hospital-based case-control study conducted in Kiambu County, Kenya. The paper is well-written and the methodology easy to follow that was used to investigate risk factors for major external structural malformations. The investigators are to be commended for evaluating risk factors for structural malformations in a developing country. This is an important step toward understanding potential risk factors for the ultimate goal of primary prevention. I have a few suggestions for the investigators to consider that may strengthen the paper.

 Methods:

1. There are inconsistencies with the specified time period of data collection/enrollment of subjects: “May 31, 2018 to and July 31, 2019”; “May to July 2019”; “three-month study period”.
2. Exposure time period should be clarified for pesticides and how the exposure determined. The investigators should also consider investigating maternal tobacco use, pre-gestational diabetes (specifically), and periconception infections.
3. The investigators conducted a sample size calculation based on all cases. The challenge with this idea is that birth defects are a very heterogeneous group and lumping them altogether suggests that their risk factors are similar. Unfortunately, this is not the case.

Results:

1. Table 3: no need to have a p value listed for the reference group.
2. Since the investigators report an increased risk for maternal age >35 years, did any of the cases have a chromosomal etiology?
3. Did affected siblings have the same type of birth defect?

Discussion - Limitations: small sample size, based on combining several different types of birth defects.

Is the work clearly and accurately presented and does it cite the current literature?

Yes

If applicable, is the statistical analysis and its interpretation appropriate?

Partly

Are all the source data underlying the results available to ensure full reproducibility?

Yes

Is the study design appropriate and is the work technically sound?

Yes

Are the conclusions drawn adequately supported by the results?

Partly

Are sufficient details of methods and analysis provided to allow replication by others?

Yes

Reviewer Expertise:

Epidemiology, birth defects

I confirm that I have read this submission and believe that I have an appropriate level of expertise to confirm that it is of an acceptable scientific standard, however I have significant reservations, as outlined above.

# Response

**Published:** 2021-04-11

**Authors:** George Agot

**Affiliations:** University of Nairobi, College of Health Sciences, School of Public Health, Kenya

**Methods**

**1. Data collection period: **This has been corrected to imply a three-month data collection period from May 31, 2019 to July 31, 2019. It was a typing error, but has since been corrected.

**2. Exposure time period for pesticides: **This variable has been defined as maternal exposure to farm-sprayed pesticides at conception. Increased likelihood of maternal exposure to pesticides was considered because agriculture is the economic mainstay in the county. ***Maternal tobacco use:*** -This variable was assessed as a variable of interest, however was dropped because only 2 out 0f 408 respondents reported its use. ***Pre-gestational diabetes: - ***This was not assessed as a variable of interest, however it was alluded to and assessed among other chronic illnesses as a proxy for measuring maternal use of teratogenic therapeutic agents for conditions such as epilepsy, depression, hypertension and diabetes mellitus. ***Infections: - ***Infection is indeed an important predictor for birth defects, however, it was beyond the scope of this study since data were gathered retrospectively. 

**3. Sample size calculation: **Birth defects are largely heterogeneous in their etiology, however the defects were lumped together for sample size calculation because of the extreme rarity of these defects coupled with unavailability of hospital-based/population-based surveillance programs in the county. Nevertheless, this has been cited as a limitation of the study.

**Results**

**1. Table 3: **P-values for binomial variables were deleted, whereas likelihood ratio test (LRT) was performed for nominal variables/variables with more than two categories to estimate the associated P-values for each variable.

**2. Maternal age:** The reference category for maternal age was changed to <35 years, but showed no association in the univariable analysis. Nonetheless, the study results were suggestive of chromosomal etiology because some cases were reported to occur with down syndrome, whereas autism was also reported by control subjects as a defect in the previous births.

 3. **Siblings with same types of birth defects:** Yes this was observed in the study. A recurrence of clubfoot was reported by some case subjects, whereas other types of birth defects were reported  by case subjects to have occurred with clubfoot

**Discussion:** Small sample sample based on combined several different types of birth defects has been cited as a limitation of this study.

# Peer review: Reviewer response for version 1

**DOI:** 10.5256/f1000research.53822.r81051

**Published:** 2021-03-16

**Authors:** Yoseph Worku

**Affiliations:** 1 Department of Public Health, St. Paul's Hospital Millennium Medical College, Addis Ababa, Ethiopia

It is interesting, technically sound and intelligibly written manuscript. 

 There are minor points to be improved. 

 In the abstract, the results and the conclusions part should show consistent interpretation and conclusion. The conclusion should base on age < 35 years. The reference is Age > 35 years (would have been better to take the <35 years as a reference). While presenting the classes, since it is dichotomized, it is better to show a common number as a margin of the classes . E.g. <35 and > 35 OR < 9 and > 9. 

 The Conceptual Framework should reflect the classifications of the risk factors presented in the Introduction. Some of the variables need to be regrouped in themes and the Framework should be redesigned accordingly.

 Some of the variables need to be defined. E.g. Pesticide exposure, chronic illness,... 

 Check the Sample Size Determination part - the Epi Info calculation does not show the same number. 

 In the univariable analyses, why p-values for the reference categories are included? 

 The discussion is a bit shallow. Comparison with more literatures, more in-depth look in to the implications and significances of the findings, and addressing also key relevant factors without significant association in the current study can improve the Discussion part. The paternal age was also mentioned as key factor in previous studies but not assessed in the current study. Why? 

 There are also other possible limitations not mentioned. E.g. survivor bias and not controlling for some relevant variables in the multivariable analysis like the paternal age. 

 The conclusion is a bit beyond the scope of the study. E.g. awareness level of couples or the community is not assessed. Detailed and in-depth discussion by citing other relevant literatures can help readers to better understand the situation and to deduce more appropriate conclusions.

Is the work clearly and accurately presented and does it cite the current literature?

Yes

If applicable, is the statistical analysis and its interpretation appropriate?

Yes

Are all the source data underlying the results available to ensure full reproducibility?

Yes

Is the study design appropriate and is the work technically sound?

Yes

Are the conclusions drawn adequately supported by the results?

Yes

Are sufficient details of methods and analysis provided to allow replication by others?

Yes

Reviewer Expertise:

Public Health and Epidemiology

I confirm that I have read this submission and believe that I have an appropriate level of expertise to confirm that it is of an acceptable scientific standard.

# Response

**Published:** 2021-04-11

**Authors:** George Agot

**Affiliations:** University of Nairobi, College of Health Sciences, School of Public Health, Kenya

**Abstract: **Conclusions have been aligned to the study results and interpretations. Maternal age <35 years has been used as the reference category, and presented as <35 years, and >= 35 years, however it was not included in the inclusion because it was no longer associated with dependent variable in the univariable analyses.

**Gestational age**: Gestational age has also been presented as <9 weeks, and >=9 weeks.

**Pesticides exposure: **Exposure redefined as maternal exposure to farm-sprayed pesticides before conception.

**Chronic illnesses: **Chronic illnesses were used as a proxy for measuring maternal use of teratogenic therapeutic agents for chronic conditions such as epilepsy, depression, hypertension and diabetes mellitus. In this respect, it was not used as a measure for particular chronic illnesses.

**Conceptual framework: **Redesigned to reflect the three classes of major external structural birth defects described in the study introduction.

**Univariable analyses:** P-values for binomial variables were deleted, whereas likelihood ratio test (LRT) was performed for nominal variables/variables with more than two categories to estimate the associated P-values for each variable.

**Discussion: **Discussion of the significant variables was improved to include their implications, and significance. Variables that showed no associations were also explained with reference to other studies.

**Paternal age: **Paternal age was introduced in the model, however it showed no association with the defects in the univariable analyses. Nevertheless, it was controlled for in the multivariable, but still showed no association. Further, because of potential collinearity with materteral age, paternal age was controlled for without maternal age in the multivariable analyses, however, still showed no association with birth defects.

**Limitations of the study: **Survivor bias was  included as a limitation of this study  because some of these defects for example neural tube defects are potentially fatal. 

**Conclusions:** Conclusions were aligned to the study findings and interpretations.

# Response

**Published:** 2021-04-11

**Authors:** George Agot

**Affiliations:** University of Nairobi, College of Health Sciences, School of Public Health, Kenya

**Sample size determination: **The sample computation was reconfirmed and found consistent with formula provided, however the hypothesized odds ratio is 2.0 (universally accepted, and not 3.0 as presented. This typing error has since been corrected.
